# Wnt Signaling in Form Deprivation Myopia of the Mice Retina

**DOI:** 10.1371/journal.pone.0091086

**Published:** 2014-04-22

**Authors:** Mingming Ma, Zhengwei Zhang, Ergang Du, Wenjing Zheng, Qing Gu, Xun Xu, Bilian Ke

**Affiliations:** 1 Department of Ophthalmology, Shanghai First People's Hospital, Shanghai Jiao Tong University, Shanghai, China; 2 Shanghai Key Laboratory of Fundus Disease, Shanghai, China; 3 Jinhua Eye Hospital, Jinhua, Zhejiang, China; Dalhousie University, Canada

## Abstract

**Background:**

The canonical Wnt signaling pathway plays important roles in cellular proliferation and differentiation, axonal outgrowth, cellular maintenance in retinas. Here we test the hypothesis that elements of the Wnt signaling pathway are involved in the regulation of eye growth and prevention of myopia, in the mouse form-deprivation myopia model.

**Methodology/Principal Findings:**

(1) One hundred twenty-five C57BL/6 mice were randomly distributed into form-deprivation myopia and control groups. Form-deprivation myopia (FDM) was induced by suturing the right eyelid, while the control group received no treatment. After 1, 2, and 4 weeks of treatment, eyes were assessed *in vivo* by cycloplegic retinoscopic refraction and axial length measurement by photography or A-scan ultrasonography. Levels of retinal Wnt2b, Fzd5 and *β*-catenin mRNA and protein were evaluated using RT-PCR and western blotting, respectively. (2) Another 96 mice were divided into three groups: control, drugs-only, and drugs+FDM (by diffuser). Experimentally treated eyes in the last two groups received intravitreal injections of vehicle or the proteins, DKK-1 (Wnt-pathway antagonist) or Norrin (Wnt-pathway agonist), once every three days, for 4 injections total. Axial length and retinoscopic refraction were measured on the 14th day of form deprivation.

Following form-deprivation for 1, 2, and 4 weeks, FDM eyes had a relatively myopic refractive error, compared with contralateral eyes. There were no significant differences in refractive error between right and left eye in control group. The amounts of Wnt2b, Fzd5 and β-catenin mRNA and protein were significantly greater in form-deprived myopia eyes than in control eyes.DKK-1 (antagonist) reduced the myopic shift in refractive error and increase in axial elongation, whereas Norrin had the opposite effect in FDM eyes.

**Conclusions/Significance:**

Our studies provide the first evidence that the Wnt2b signaling pathway may play a role in the development and progression of form-deprivation myopia, in a mammalian model.

## Introduction

The extraordinarily high prevalence of myopia in Asian countries and regions such as China, Singapore, Hong Kong, and Japan has become an important public health problem [1], [2]. Many blinding diseases such as retinal detachment, pigmentary degeneration, myopic macular degeneration, glaucoma and cataract have a potential association with pathologic myopia [3]–[5]. Recently, many experimental myopia models have been established, and some signaling pathways and targets in regulating the development of myopia have been investigated [6]–[12]. Retinoic acid [13], [14], dopamine [15], [16], bFGF [17]–[19], vasoactive intestinal polypeptide [20], [21], insulin-like growth factor-1 [22], [23], serotonin [24] and melatonin [25] have been shown to be involved in the development of myopia. More generally, four signaling pathways are currently recognized as particularly important in the development of body tissues and organs (BMPs, Hedgehogs, FGFs, and Wnt). The first three of these have been implicated by research findings in the development of myopia [26]–[30]. However, whether or not Wnt signaling contributes to the development of myopia has not been determined.

The canonical Wnt signaling pathway is a critical regulator of tissue patterning and regeneration, embryogenesis and cancer development [26]. Some retinal diseases such as familial exudative vitreoretinopathy, retinitis pigmentosa and Norrie's disease are caused by changes in the Wnt signaling pathway [31]. Furthermore, the canonical Wnt signaling pathway is also a key pathway in regulating development of the eye in the embryonic period. It was found that functional expression of this pathway in the mature retina could regulate ocular development [32]–[38]. The Wnt/*β*-catenin signal has been shown to be a key regulator of various stages, including vasculogenesis in the retina, maintenance of retinal stem cell progenitors and neuronal specification, development of the cornea and the lens, and formation of the ciliary body [26], [39]. Wnt2b, which is expressed in the equatorial margin of the developing retina, is a Wnt family protein that has been shown to control differentiation of the progenitor cells in the retina. Frizzled 5 is a Wnt receptor expressed in the retina. After blocking either Fzd5 or Wnt/*β*-catenin signaling within the developing retina, cell proliferation was reduced and the onset of proneural gene expression was inhibited [40]. In a genetic knock-out experiment, obvious signs of hypoplasia– including increased cell death in the ventral retina, delayed and/or incomplete closure of the ventral fissure, an excess of mesenchymal cells in the vitreous cavity, and persistence of the hyaloid vasculature in association with a large number of pigment cells– were detected in Wnt2b knockout mice or Fzd5 knockout mice [41]–[43]. Fzd5 knockout mice also exhibit a late-onset progressive retinal degeneration by ∼6 months of age [43].

In the present study, we performed two separate experiments to test the hypothesis that the Wnt/*β*-catenin signaling pathway plays an important role in myopia development. Experiment I: investigate the potential trend of Wnt2b pathway in form-deprivation myopia (FDM) eyes induced by eyelid suture. Experiment II: investigate the possibility of a regulatory role for the Wnt2b pathway in eye growth and FDM (induced by wearing a diffuser).

## Materials and Methods

### Reagents

TriZol was purchased from Invitrogen (NY, US). Reverse Transcription and RT-PCR System were purchased from Roche (Mannheim, Germany) and Takara (Shiga, Japan), respectively. All other reagents were obtained from Sigma (St. Louis, MO). For western blot analyses and immunocytochemistry, a rabbit polyclonal antibody against the humanWnt2b receptor (ab50575), a polyclonal antibody against human Fzd5 (ab14475) and a monoclonal antibody against β-catenin (E247) (ab32572) were obtained from Abcam (MA, US), and an antibody to rat GAPDH was obtained from Epitomics (2251-1, CA, US). The HRP-conjugated secondary antibody (sc-2004) for western blotting was from Santa Cruz Biotechnology (CA, USA). The proteins DKK-1 (a Wnt-pathway inhibitor) and Norrin (a Wnt-pathway activator) were purchased form R&D Systems (Minneapolis, MN, USA).

### Animals

Male three-week-old C57BL/6 mice were obtained from the Shanghai Laboratory Animal Center (Shanghai, China). All animals were examined clinically for confirming the corneal transparency of each eye and no injuries or infections of the eyes. Mice were housed in a 12-h light-dark cycle. Refractive errors were induced monocularly by suturing the right eyelids in one group of mice in experiment I or wearing a translucent diffuser on the right eye in experiment II, which was named the FDM group. The left untreated eyes in the FDM mice served as a contralateral eye control group. Untreated mice provided normal control data for refractive errors, which was named the control group. The experimental protocols used in this study followed guidelines established by the ARVO Statement for the Use of Animals in Ophthalmic and Vision Research and were approved by the Ethics Committee of Shanghai First People's Hospital, Shanghai Jiaotong University, Shanghai, China (Permit Number: 2009-0086).

### Refraction Measurement

With the animal restrained and in the dark, the refractive state was measured by streak retinoscopy at 50 cm working distance, using lens bars to neutralize on the 2 principal meridians. In mice, the ciliary muscle is small and has a cylindrical shape; the small size of the muscle and the large size of the lens are consistent with the reported absence of accommodation in mice [44]. Despite the apparent lack of need for cycloplegia, however, tropicamide (Santen Pharmaceutical Co., Ltd) was also used before measurement – to dilate the small pupil, and thus lessen the difficulty of refracting. Reliability of these measurements was good, according to the intraclass correlation coefficient (0.76, 95% CI: 0.6–0.8) computed after five replicate measurements of the same eye in 20 different animals; carried out in a masked fashion. All refraction data were reported as mean spherical equivalents. Three replicate measures of the same eye were taken. We recognize that, because of the small-eye artifact, [45] these measurements cannot be regarded as giving correct absolute magnitude of refractive error, but they are still reliable for comparing relative refractive error within the same animals or among a cohort of animals treated at the same time.

## Experiment I: Expression of Wnt2b Pathway in Normal and FDM Eyes Induced by Eyelid Suture

### Establishment of Form-Deprivation Myopia

Right eyes were used as the experimental eyes in the Experiment I. Treatments commenced for right eyelid suture during post-natal week 3. The groups and durations of lid suture were: 1 week (n = 25); 2 weeks (n = 25) and 4 weeks (n = 30). The groups and durations of controls were: 1 week (n = 15); 2 weeks (n = 15) and 4 weeks (n = 15). After anesthetizing with an i.p. injection of a xylazine/ketamine mixture (xylazine 2 mg/kg + ketamine 50 mg/kg), the eyelids of the right eyes were simply sutured with 5° nylon non-absorbable sutures.

### Axial Length Measurement

In Experiment I, the axial length of the eye was measured by two different methods: A-scan and photographic imaging. A-scan ultrasonography (KN-1800) with a 10-MHz transducer was used when the animals were anesthetized. The axial length of the eye was defined as the distance from the front of the cornea to the back of the sclera.

After measurement by A-scan, photographic imaging was used as an alternative method to confirm the measurement of axial length. After euthanasia by 5% isoflurane inhalation, the eyes were immediately enucleated and put under an operating microscope. Photos were taken through the eyepieces of the microscope, and the axial length of the eye was measured in magnified photos, where one pixel of the image corresponded to 14 µm. The reliability of these measurements was evaluated according to the intraclass correlation coefficient (axial length measurement: SD = 19.6 µm, 95%CI = 5.3–31.7 µm), computed after three replicate measurements of the same eye in five different animals, obtained in a masked fashion by two observers.

### RNA Isolation and RT-PCR

To detect the natural developmental trends of Wnt2b, Fzd5 and β-catenin mRNA expression in normal mice, 36 untreated mice were used in this set of experiments. After an overdose of anesthetic, retinas from both eyes were dissected and pooled as a separate sample at 3, 4, 5 and 7 weeks after birth. Nine mice were used at each time.

In order to examine the changes in expression of Wnt2b, Fzd5 and β-catenin mRNA during FDM, retinas from each group were dissected at the given time point (1, 2 and 4 weeks after lid suture). Each treated and each control retina, from each mouse, was handled as a separate sample, and 10 FDM mice and 6 control mice were used at each time. RNA was isolated using TRIzol according to the manufacturer's instructions. One microgram total RNA was used for complementary DNA (cDNA) synthesis using the Reverse Transcription System according to the manufacturer's instructions. cDNA was amplified by PCR using primers specific for mouse Wnt2b, Fzd5, β-catenin, and β-actin in a thermal cycler (PCR Sprint; Thermo Hybaid, US). The sequences of the primers were as follows: Wnt2b sense 5^′^-CTG CAC GCT CTT GGG AAC-3^′^ and antisense 5^′^-ATA GCT CCC CGC AGA CTC C-3^′^ ; Fzd5 sense 5^′^-GGC GAA AGA GTG CGA GAG-3 and antisense 5^′^-TCA CAG CAC AGC GAG CAG-3^′^ ; β-catenin sense 5^′^-GGC AGC GGC AGG ATA CAC-3^′^ and antisense 5^′^-TTC ACA GGA CAC GAG CTG A-3^′^ ; β-actin sense 5-CAC TGC CGC ATC CTC TTC CTC-3 and antisense 5^′^-TGC TGT CGC CTT CAC CGT TCC-3^′^ . Primers were designed by using the Primer-BLAST primer-design tool at NCBI (http://www.ncbi.nlm.nih.gov/tools/primer-blast/index.cgi?LINK_LOC=BlastHome). β-actin served as the internal control. Each PCR reaction contained 0.5 µM primers, 200 µM dNTPs, 1.5 µM MgCl_2_, 1.25 U of Taq polymerase, and one microliter cDNA. The parameters were set as follows: 37°C/1 h; 95°C/5 min and 45 amplification cycles of 95°C/5 s; 60°C/20 s. Relative mRNA was normalized to β-actin and fold-changes were calculated using the 2^−ΔΔCt^ method as described previously [46].

### Western Blot Analyses

Fourteen FDM mice and 9 control mice at each time were used in this part of experiment. To ensure that sufficient amounts of protein were obtained, dissected retinas from 2 mice were pooled as a separate sample in the FDM group or contralateral eye group. Both retinas from one mouse were dissected and pooled as a separate sample in the control group.

Nuclear protein extraction, to measure the amount of β-catenin protein, was done as follows: Dissected retinas were washed with PBS (phosphate-buffered saline, 0.01 M, pH 7.4) in triplicate, and nuclear protein was extracted in buffer (20 mM Hepes pH 7.9, 420 mM NaCl, 1.5 mM MgCl_2_ , 0.2 mM EDTA, 25% glycerol and 0.5 mM DTT) containing protease inhibitors (1 mM phenylmethylsulfonyl fluoride, aprotinin 30 µL/mL, and sodium orthovanadate 100 nM), as described before [47]. Retinas were homogenized by sonication and incubated with shaking at 4°C for 30 min. The mixture was then centrifuged (16,000×g at 4°C for 5 min), and the supernatant was isolated as a nuclear fraction of proteins. This fraction was used to quantify the levels of β-catenin protein.

Total protein extraction for measurement of Wnt2b and Fzd5 proteins: Dissected retinas were washed with PBS in triplicate, and total proteins were extracted in RIPA buffer (10 mM Tris-HCL pH 7.4, 150 mM NaCl, 1% deoxycholic acid, and 1% Triton X-100), containing protease inhibitors (1 mM phenylmethylsulfonyl fluoride, aprotinin 30 µL/mL and sodium orthovanadate 100 nM). Retinas were homogenized by sonication and centrifuged at 2000×g for 15 min at 4°C. Proteins in the supernatant were the total soluble protein, which was then used to quantify the levels of Wnt2b and Fzd5 proteins.

Measurement of extracted protein content by quantitative immunoblotting: Proteins were separated using 10% sodium dodecyl sulfate-polyacrylamide gel electrophoresis (SDS-PAGE) and transferred onto polyvinylidene fluoride (PVDF) membranes. The membranes were blocked for 2 h at room temperature in 5% nonfat dried milk in a buffer containing 10 mM Tris-HCL (pH 8.0), 150 mM NaCl, and 0.05% Tween-20, and then incubated with a primary antibody for 2 h at room temperature or overnight at 4°C at the following dilutions: anti-Wnt2b antibody at a dilution of 1∶1000 in Tris Buffered Saline with Tween 20 (TBST) containing 137 mM Sodium Chloride, 20 mM Tris, 0.1% Tween-20, anti-Fzd5 antibody at 1∶1000, anti-β-catenin antibody at 1∶5000, and β-actin antibody at 1∶1000. The membranes were washed in triplicate with TBST and incubated with HRP-conjugated secondary antibody at a dilution of 1∶5000 for 2 h. Finally, the membranes were washed in triplicate with TBST and developed using the enhanced chemiluminescence method. The density of the protein of interest on the film was measured using densitometric measurement. All densities above threshold and below saturation were included. Density measurements were then normalized to β-actin readings.

## Experiment II: Effects of DKK-1 and Norrin on FDM (Translucent Diffuser) and Non-FDM Eyes

### Establishment of Form-Deprivation Myopia

A translucent diffuser was worn on the right eye to induce FDM in 36 age-matched mice. After anaesthetizing with i.p. xylazine/ketamine, frosted hemispherical thin plastic shells, as described by Schaeffel et al. [45], were attached over the right eye. The diffusers attenuate light by about 0.5 log units (30% transmission). The rims, 1 mm in diameter, were affixed firmly to the fur around the eye with cyanoacrylic glue. Paws were wrapped with adhesive tape to prevent the mice from removing the diffusers, and the mice were checked 3 times per day to ensure that the diffusers were in place. Before intravitreal injection, mice were anaesthetized and diffusers were removed gently. The diffuser was reattached after each injection.

### Axial Length Measurement

In Experiment II, the axial length of the eye was measured by A-scan ultrasonography (KN-1800). The method was the same as that described above.

### Intraocular Drug Administrations

To investigate the effects of Wnt signaling and its antagonist or agonist on the development of eyes in FDM and controls, FDM was induced in C57B/L6 mice using a diffuser as described above. Ninety-six mice were randomly divided into 3 groups: control (n = 24), drugs only (n = 36) and drugs+FDM (n = 36). Twelve of the control mice received no manipulation, and the other 12 received sham-injection only in the right eye (Table 1). The right eyes of all mice in the drugs+FDM and drugs-only groups were injected with Dickkopf-1 (DKK-1) or Norrin protein in vehicle, or vehicle alone (Table 1). Intravitreal injections (3 µl) were performed with a 30-gauge needle and a microinjector (Shanghai Anting Wei Liang Jin Ye Qi factory, Shanghai, China), 0.5 mm posterior to the temporal limbus, angled toward the optic nerve until the tip of the needle was visualized in the center of the vitreous. The needle was kept in the vitreous chamber for about 30 s to ensure the full diffusion of the drugs. Ofloxacin eye ointment (Shenyang Xingqi Pharmaceutical Co., Shenyang, China) was applied after injection. Intravitreal drug administration began on the second day of occlusion and was repeated every three days for a total of four times (2nd, 5th, 8th, 11th days of FDM). Levofloxacin Hydrochloride Eye Drops 0.5% (Santen Pharmaceutical, Noto, Japan) were applied to the ocular surface before injection. A drop of 0.4% Oxybuprocaine Hydrochloride Eye Drops (Santen Pharmaceutical, Osaka, Japan) was used for additional topical anesthesia. The diffuser was reattached after each injection. Retinoscopic refraction and axial length measurement were performed on the 14th day of FDM. Recombinant mouse DKK-1 or Norrin proteins (R&D Systems, Minneapolis, MN, USA) were dissolved in PBS. A total of 3 µl of DKK-1 (10 ng/µl) or Norrin (5 ng/µl) was injected into the vitreous chamber. Axial length and refractive error were measured in 18 mice injected with DKK-1, 19 mice injected with Norrin, and 22 mice injected with vehicle. Mice that developed cataract, vitreous hemorrhage, or retinal detachment as a result of the injections, were excluded (Table 1). Measurements were performed 3 d after the last injection (14th day of form deprivation).

**Table 1:** Grouping and Exclusion of Mice Injected With DKK-1 or Norrin.

Groups	Injection	Tested	Included	Exclusion				
				Dense cataract	Vitreous hemorrhage	Retinal detachment	Death	Total
Control	No manipulation	12	12	0	0	0	0	0
	Sham-injection*	12	12	0	0	0	0	0
Drugs only	Vehicle	12	10	2	0	0	0	2
	DKK-1	12	10	1	1	0	0	2
	Norrin	12	9	1	1	0	1	3
Drugs + FDM (by wearing diffuser)	Vehicle	12	12	0	0	0	0	0
	DKK-1	12	8	2	0	1	1	4
	Norrin	12	10	2	0	0	0	2
Total		96	83	8	2	1	2	13

*Sham-injection: needle penetration and withdrawal, without injection.

### Data Analyses

Comparisons between deprived eyes and contralateral eyes in FDM mice were made using a paired *t*-test. Independent *t*-tests were used to compare FDM eyes and control eyes. Two-way ANOVA (analysis of variance) was used to make comparisons between data for each parameter at different times, after confirming the normal distribution of data. Data are represented as mean ± standard deviation (SD) in the text, or mean ±standard error of the mean (SEM) in the figures. *P*<0.05 was considered statistically significant. All statistical routines were used as implemented in SPSS version 17.0.

## Results

### Refraction and Axial Length

As shown in Table 2, the results showed that after form-deprivation treatment of 1, 2, and 4 weeks in FDM group, the differences in refraction between right eye (FDM) and left eye (contralateral non-deprived eyes) were statistically significant (paired *t*-test). There were no statistically significant differences between right and left eyes (paired *t*-test) in the control group.

**Table 2:** Refractions and Axial Lengths of FDM and Control Eyes.

			Refraction (D)				Axial Length (mm)			
Group	Treatment Druation (Days)	Number of Animals	Right Eye	Left Eye	Difference	*P*	Right Eye	Left Eye	Difference	*P*
FDM (Lid-Suture)	7	25	7.01±3.64	9.82±3.52	2.75. ±2.21	<0.001	2.84±0.01	2.85±0.02	0.01±0.01	0.57
	14	25	5.20±2.74	9.40±1.78	4.15±1.21	<0.001	2.92±0.03	2.89±0.01	0.03±0.02	0.02
	28	30	2.81±3.05	7.82±2.32	4.94±2.26	<0.001	2.98±0.01	2.90±0.01	0.07±0.01	<0.001
Control	7	15	10.21±2.87	10.34±3.25	0.12±1.79	0.82	2.83±0.02	2.84±0.01	0.01±0.01	0.76
	14	15	9.66±2.82	9.54±1.28	0.11±2.01	0.75	2.89±0.01	2.88±0.01	0.01±0.02	0.5
	28	15	8.13±1.74	8.05±2.79	0.09±1.23	0.52	2.92±0.01	2.93±0.02	0.01±0.01	0.59

FDM: form-deprivation myopia.

Data are presented as mean±SD.

Axial length was measured by A-scan.

Axial length was measured by two different methods at selected times from week 1 to weeks 4. Data in table 2 were derived from A-scan measurements. Lid suture was found to induce axial elongation and differences in axial length, compared to those in experimental control eyes of magnitude, depending on the duration of unilateral lid suture. The measured axial lengths were longer in experimental eyes than in contralateral eyes after treatment of 1, 2, and 4 weeks. At 1 week of lid suture the difference in amount of axial elongation was not significant, but it increased significantly after continuation of treatment for 2 and 4 weeks (Table 2; paired *t*-test). In the control group, there were no significant differences between right and left eyes (paired *t*-test).

**Figure 1:**
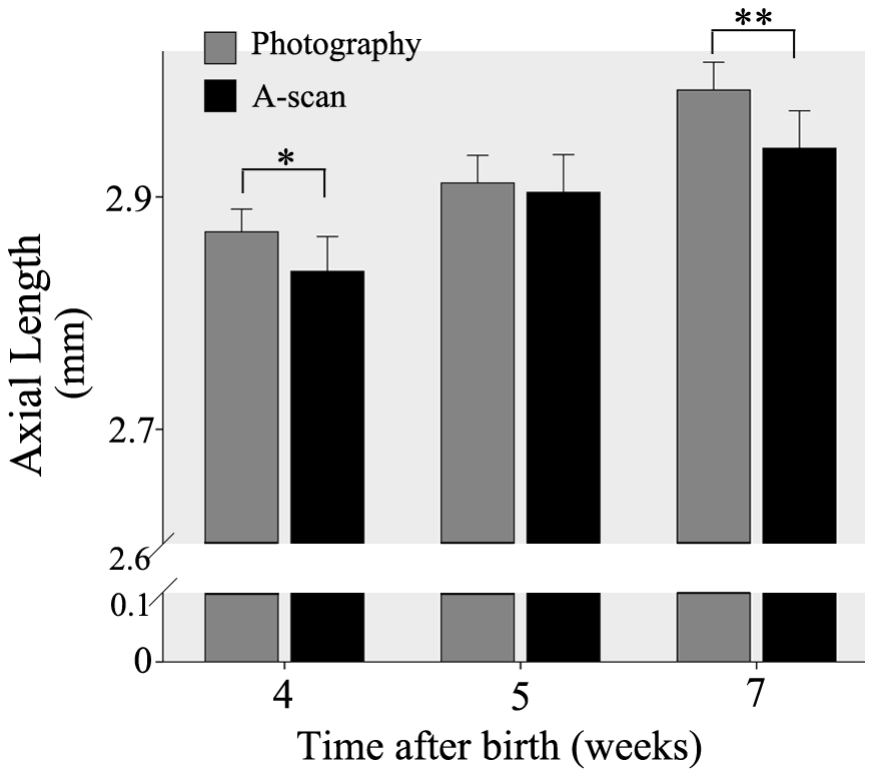
Comparison of axial length measurements by A-scan ultrasonography and by photographic imaging in control mice. The axial length measured by A-scan was less than that by photography, at 1 and 4 weeks of age. Six mice (12 eyes) were measured at each age. Data are presented as mean±SEM. (* *P*<0.05 and ** *P*<0.01).

Comparisons between the two measurement methods were made in the control group. The axial length of both eyes measured by image photography was generally slightly higher (0.04±0.03 at 1 week; 0.02±0.02 at 2 weeks; 0.07±0.04 at 4 weeks) than that measured by A-scan. There were significant differences between the two groups at 1 and 4 weeks (Fig. 1; two-way ANOVA).

### Expression of Wnt2b, Fzd5 and β-catenin mRNA in Retinas of Normal Mice

**Figure 2:**
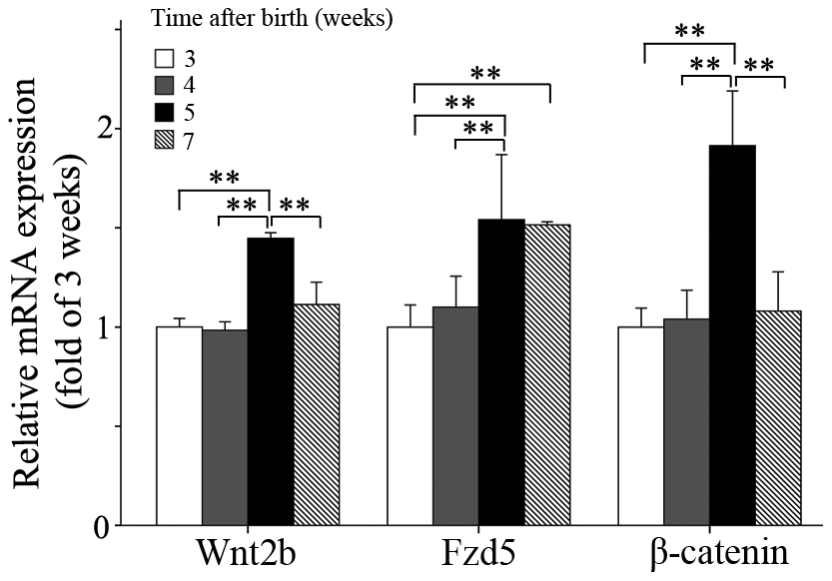
Natural trends of retinal Wnt2b, Fzd5 and *β*-catenin mRNA expression within 7 weeks after birth in normal mice. The expression level of the 3-week mice was defined as 1, and the values were normalized to those of *β*-catenin mRNA levels. Retinas from both eyes of 9 mice were used at each age. Data are presented as mean±SEM. (*P<0.05 and **P<0.01).

In mice, the Wnt/*β*-catenin pathway plays a critical role in the development of the retina, whose spatial vision is not mature until at least 4 weeks after birth [48]. Therefore, as might be expected, we found that the expressions of Wnt2b, Fzd5 and *β*-catenin mRNA were variable during the 7 weeks after birth, as shown in Fig. 2. The expression of Wnt2b mRNA increased gradually within 5 weeks after birth (two-way ANOVA), but decreased observably after 5 weeks (two-way ANOVA); that of Fzd5 mRNA increased from 3 weeks to 5 weeks following birth (two-way ANOVA) but decreased slowly after 5 weeks, and that of *β*-catenin mRNA increased gradually within 5 weeks following birth (two-way ANOVA) but decreased observably after 5 weeks (two-way ANOVA).

### Form-Deprivation Increases mRNA Expression of Wnt2b, Fzd5 and *β*-catenin

**Figure 3:**
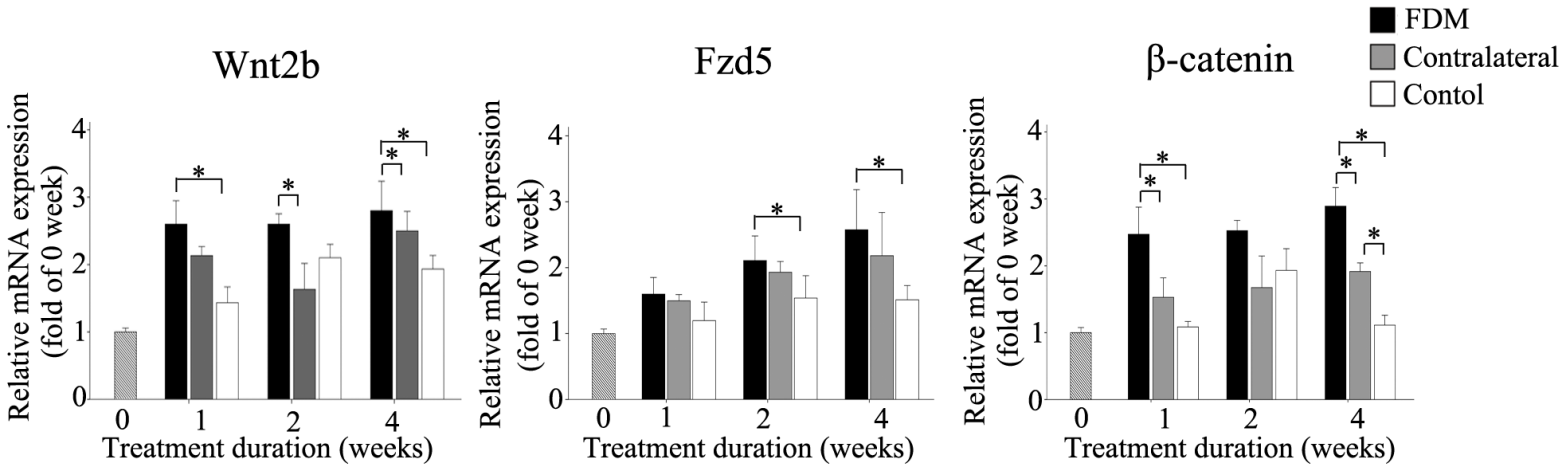
Expression of Wnt2b, FZD5 and *β*-catenin mRNA in mouse retinas as a function of treatment duration. Wnt2b mRNA was greater in the FDM than in the control group, after 1, 2 and 4 weeks of treatment; Fzd5 mRNA also was significantly greater in FDM than control, but only after 2 and 4 weeks of treatment, and *β*-catenin mRNA was greater in the FDM group than controls, but only after 1 and 4 weeks. Differences were also significant between FDM eyes and contralateral eyes at 1 and 4 weeks, and between contralateral and control eyes at 4 weeks. The expression level at week 0 was defined as 1, and the values were normalized to those of *β*-catenin mRNA levels. Retinas from both eyes of ten FDM mice and 6 control mice were used for each treatment duration. Data are presented as mean±SEM.(*P<0.05).

To investigate the changes of the Wnt2b signaling pathway in FDM mice, we quantified the expression of Wnt2b, Fzd5 and *β*-catenin mRNAs in the retinas of FDM mice. As shown in Fig. 3, the expression of Wnt2b mRNA was significantly greater in the FDM group than in the control group at 1and 4 weeks (two-way ANOVA), and significant differences were found between FDM and contralateral non-deprived eyes (two-way ANOVA) at 2 weeks. The expression of Fzd5 mRNA was significantly greater in the FDM group than in the control group at 1 and 2 weeks (two-way ANOVA). The expression of *β*-catenin mRNA was significantly greater in the FDM group than in the control group (two-way ANOVA), as well as to that in the contralateral eyes (two-way ANOVA), at 1 and 4 weeks. The expression of *β*-catenin mRNA was also significantly greater in retinas contralateral to FDM eyes, than in control animals, at 1 and 4 weeks of treatment. (Fig. 3, right)

### Form-Deprivation Increases Retinal Content of Wnt2b, Fzd5 and *β*-catenin Proteins

**Figure 4:**
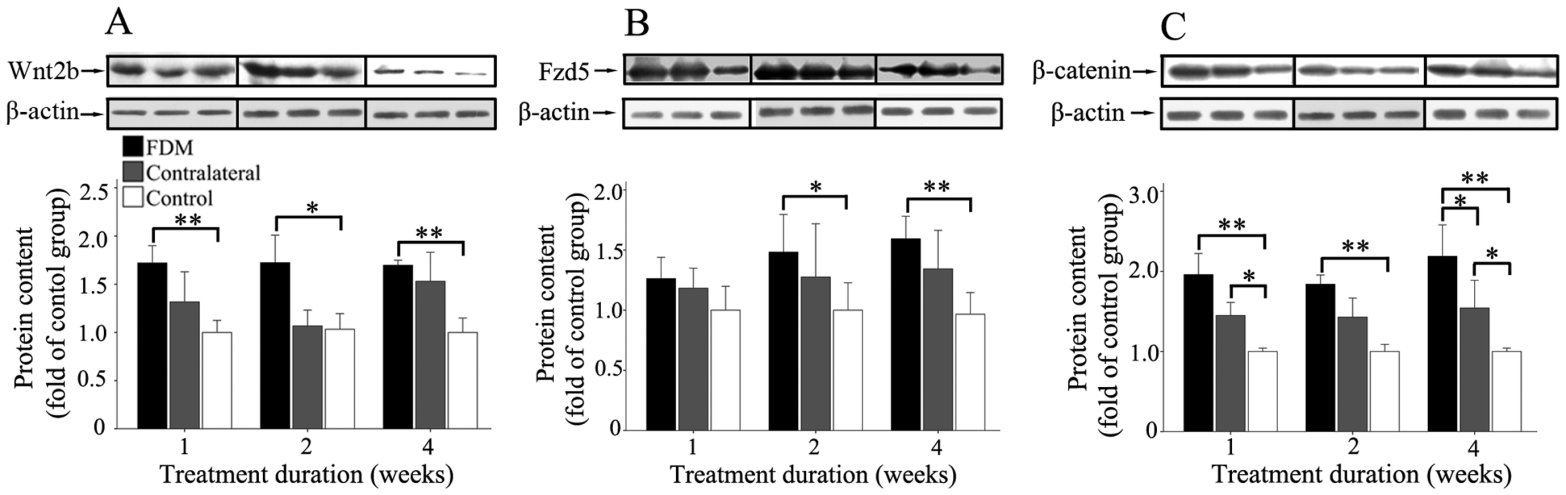
Form deprivation increased the retinal content of Wnt2b, Fzd5 and β-catenin proteins. (A) Wnt2b protein content in the FDM group was significantly greater than that in the control group, at 1, 2 and 4 weeks. (B) Fzd5 protein content in the FDM group was significantly greater than that in the control group, at 2 and 4 weeks. (C) Nuclear *β*-catenin protein content was significantly higher in the FDM group than in the control group at 1, 2 and 4 weeks. Significant differences between FDM eyes and contralateral eyes also were found at 4 weeks, and content was significantly higher in the contralateral eye group than in the control group at 1 and 4 weeks. Retinas from both eyes of fourteen FDM mice and 9 control mice were used for each treatment duration. Data are presented as mean±SEM. (* *P*<0.05 and ** *P*<0.01).

We investigated the levels of Wnt2b, Fzd5 and *β*-catenin proteins in retinas of FDM mice, using western blotting. As shown in Fig. 4, Wnt2b protein content was significantly greater in the FDM group than in the control group (two-way ANOVA), after treatment for 1, 2, and 4 weeks. Fzd5 protein content was significantly greater in the FDM group than in the control group (two-way ANOVA), only after treatment for 2 and 4 weeks. *β*-catenin protein content in the nuclear fraction was significantly greater in the FDM group than in the control group (two-way ANOVA), after treatment for 1, 2, and 4 weeks; it was also significantly greater in the FDM group than in the control group at 4 weeks, and significantly greater in the contralateral group than in the control group at 1 and 4 weeks (two-way ANOVA).

### The Wnt/*β*-catenin Pathway in the Eye Is Involved in the Development of FDM

**Figure 5.Wnt:**
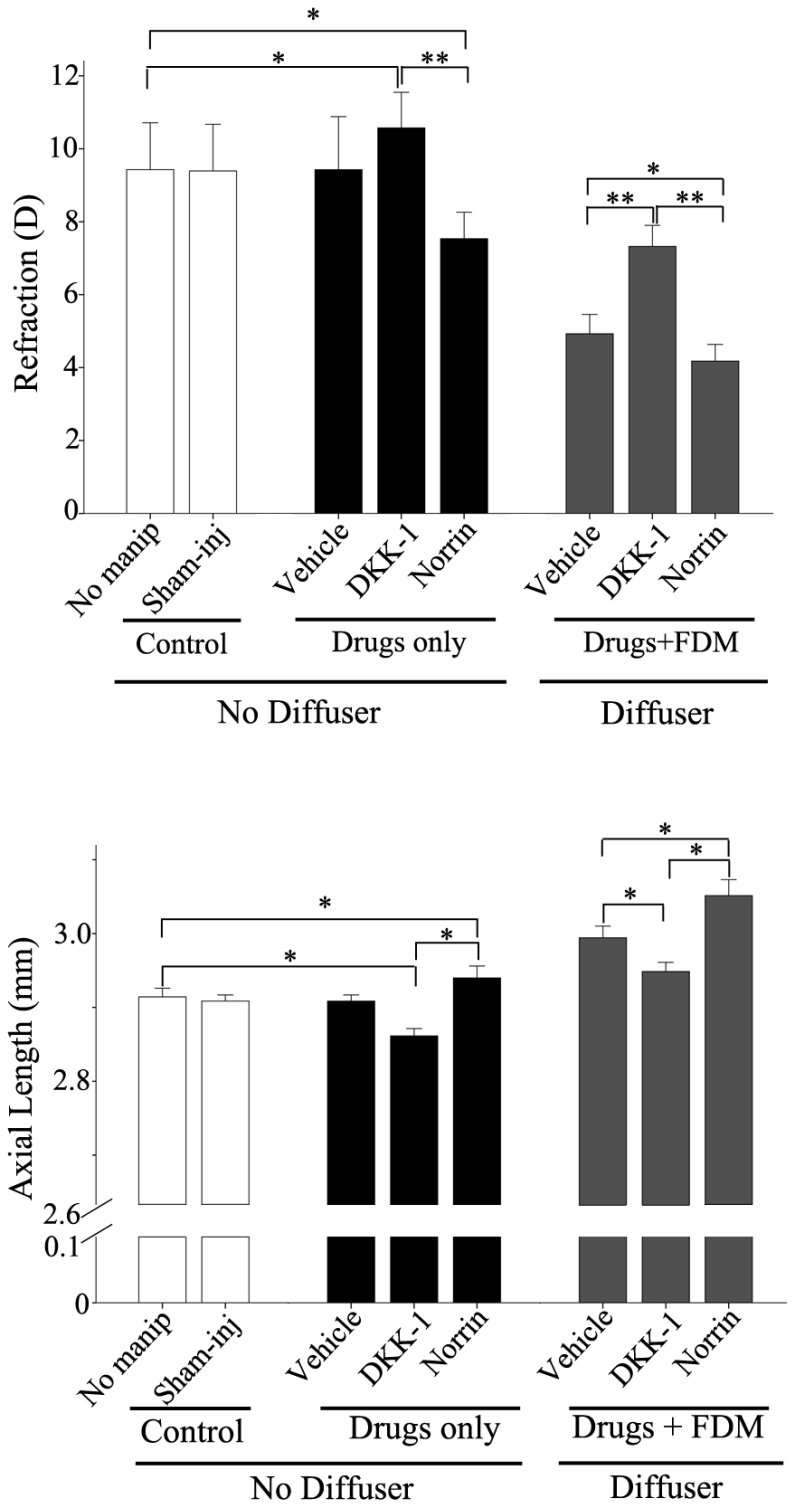
pathway is involved in the development of FDM. Intravitreal drug administration began on the second day of occlusion and was repeated every three days for a total of four times (2nd, 5th, 8th and 11th days of FD). A total of 3 µl of DKK-1 (10 ng/µl) or Norrin (5 ng/µl) was injected into the vitreous chamber each time. Refraction and axial length were measured 3 days after the last injection. There were no differences, neither between no-manipulation (“no-manip” in the figure) and sham-injection (“sham-inj” in the figure), nor between these and injection with vehicle in the drugs-only group. DKK-1 significantly reduced the time-dependent increases in axial length, and significantly increased refractions (made them more positive or hyperopic), while Norrin had the opposite effect. Six mice were used in each group. Data are presented as mean±SEM.(* *P*<0.05 and ** *P*<0.01).

In order to test for a possible role of the Wnt/*β*-catenin pathway in the development of FDM, we delivered a Wnt-pathway agonist or antagonist to the retina by intraocular injection. Norrin is a Wnt-like protein that binds with high affinity to the receptor Fzd and *activates* the canonical Wnt signaling pathway. DKK-1, a member of the DKK (Dickkopf) family, is a well-documented *antagonist* of this pathway. In *non-deprived eyes*, there were no differences in the development of FDM between mice receiving no manipulation and control mice receiving needle-penetration only (sham-injection) or vehicle (solvent) injection only. Norrin significantly stimulated axial growth and caused a myopic (negative) shift in refraction, whereas DKK-1 had the opposite effect (Table 3, Fig. 5; two-way ANOVA). In *form-deprived (diffuser) eyes*, the effects of these two agents were similar, except that in FDM the values were offset by the negative shift in refraction and the excess in axial length, induced by the diffuser. When the Wnt pathway was activated with Norrin or inhibited with DKK-1, refraction and axial length were different from those in the “no manipulation” control group (Table 3, Fig. 5; two-way ANOVA).

**Table 3:** Effect of DKK-1 or Norrin on the Refraction and Axial Length.

	Control		Drugs only			Drugs + FDM (by wearing diffuser)		
	No manipulation	Sham-injection	Vehicle	DKK-1	Norrin	Vehicle	DKK-1	Norrin
Refraction (D)	9.42±1.16	9.40±1.07	9.40±1.22	10.55±0.97	7.55±1.27	4.95±1.12	7.32±1.20	4.16±1.02
Axial Length (mm)	2.91±0.03	2.91±0.01	2.92±0.02	2.84±0.01	2.95±0.02	2.99±0.02	2.93±0.03	3.05±0.03

Data are presented as mean±SD.

## Discussion

The Wnt pathway is a very important signaling pathway, which plays critical roles in the development of body tissues and organs, yet studies on its possible relationship to the cause and prevention of myopia have not yet been reported. In the present study, first we detected significant increases in mRNA and protein markers for Wnt2b signaling in the retinas of FDM eyes, and then we found that refraction and axial length were altered by intravitreal injection with DKK-1 or Norrin. The former suggested that changes in the canonical Wnt pathway were plausible candidates as regulators of ocular growth and refraction in mice, and the latter provided concrete *in vivo* evidence that the Wnt2b signaling pathway is in fact such a regulator. Thus we have shown for the first time that the Wnt signaling pathway is likely to play some role in the development and prevention of myopia.

The successful establishment of a myopia animal model is the basis of investigating the relationship between Wnt signaling pathway and myopia. Various experimental animal myopia models have been used in previous studies, such as chicks, guinea pigs, tree shrews, and marmosets [8]–[14]. In the present study, we chose to use C57BL/6 mice because of the better genetic mapping, wide range of use, simple and convenient operation and high success rate in these animals. After suturing the eyelids of the right eye, the differences in refraction between deprived (FD) eyes and contralateral non-deprived eyes were significant after 1, 2 and 4 weeks, and the differences in axial length were significant after 2 and 4 weeks. All of these indicated a successful establishment of the mouse myopia model, and in particular, the validity of our methods for detecting and quantifying the small changes in eye length and refraction due to form-deprivation in the mouse. Axial length measured by A-scan ultrasonography was slightly but significantly shorter than axial length measured in photographic images. Given that the instrumental resolution of the two methods is similar, we suggest that the A-scan measurements are more accurate; because they are obtained *in vivo*, they are not subject to *ex vivo* distortion – such as slight flattening and axial elongation –that may occur when the excised eye is laid on its side for photography. However, it is also possible that the axial length values inferred from A-scan ultrasonography are in error, because of incorrect assumptions about the speed of sound in different tissues along the visual axis.

The canonical Wnt signaling pathway is a key regulator of tissue patterning, embryogenesis and regeneration, and in the eye has been shown to be a key regulator of various stages of retinal development, including retinal field establishment, maintenance of retinal stem cells, neuronal specification, vasculogenesis in the retina, and formation of the ciliary body [26], [39]. Wnt2b protein was detected in the whole human retina at E12.5 week (embryo), and in the amacrine cells of the inner nuclear layer and retinal ganglion cells at 1 week following birth and in mature retinas [35], [37]. In our study, expression of Wnt2b, Fzd5 and *β*-catenin mRNAs in normal mice showed a notable trend of “rise first and then fall”, from 3 weeks to 7 weeks after birth: increasing significantly to a peak at 5 weeks after birth, then decreasing significantly from 5 to 7 weeks after birth. In the FDM group, in contrast, Wnt2b mRNA and protein content increased and remained at higher levels than in the control group from 3 weeks to 7 weeks after birth. This trend of “rise first and then fall” in normal mice disappeared in the FDM eyes, as form-deprivation activated the Wnt signaling pathway and maintained it at a high level. Furthermore, the up-regulation in Wnt2b expression increased with the expression of Fzd5. The changes in *β*-catenin mRNA and protein content were similar to those in Wnt2b and Fzd5 in normal mice, whereas those in the FDM group increased and remained higher than in the control group from 1 week to 4 weeks of treatment. This showed that the Wnt signaling pathway was activated during the development of FDM.

In the natural development of a normal mouse, the trend of “rise first and then fall” in the Wnt signaling pathway not only ensures the normal development and growth of the eyes from 3 to 7 weeks after birth, but also prevents excessive growth of the eyes. This trend is similar to the trend in retinal expression of growth-associated protein-43 (GAP-43 or B50), which is important to the development and regeneration of neurons [49]–[51]. In our study, the Wnt signaling pathway was activated abnormally in FDM mice retinas from the first week of eyelid suture and maintained at a high level, but did not decrease at the fifth postnatal week as it did in normal mouse retinas. These observations are consistent with the idea that failure of inhibition of Wnt signaling in the retina may promote the development of myopia.

In our study, the content of Wnt2b protein in the retina was significantly increased in the FDM group. Fzd5, a member of the seven-transmembrane-domain Fzd receptor family, is a receptor for the Wnt signaling pathway, which is expressed widely throughout the retina from E 8.5 day to birth [52]–[54], and in the Müller cells and amacrine cells in mature retinas [43]. Higher content of Fzd5 protein was detected in the FDM group than in the control group. β-catenin is an essential component of the canonical Wnt signaling pathway. Upon Wnt pathway stimulation, *β*-catenin becomes stabilized and translocates to the nucleus, leading to the activation of Lef-1, a HMG-box transcription factor, and gene transcription. In this study, we found that nuclear *β*-catenin protein content was significantly greater in the FDM group than in the normal control.

There is no previous report about whether Wnt signaling pathway is involved in the development of FDM. Our study revealed that activation of the canonical Wnt signaling pathway could promote the progression of myopia in mice. The effects of intravitreal injection with DKK-1 or Norrin support our hypothesis, that the Wnt pathway is involved in the development of FDM. Some genome-wide association studies (GWAS) of human myopia have indicated that Wnt signaling pathway may be associated with myopia, which could support our study [55]–[57].

We are aware of limitations to this study. Notably, further studies using transgenic mice should be taken to examine mechanisms following activation of the Wnt pathway. Nevertheless, our results on the potential relationship between FDM and the Wnt signaling pathway clearly suggest that this pathway is a potential target for preventing and treating the progression of myopia.
